# Unraveling the Role of Histone Variant CENP-A and Chaperone HJURP Expression in Thymic Epithelial Neoplasms

**DOI:** 10.3390/ijms23158339

**Published:** 2022-07-28

**Authors:** Georgia Levidou, Konstantinos Palamaris, Alexandros G. Sykaras, Georgios Andreadakis, Christos Masaoutis, Irene Theochari, Penelope Korkolopoulou, Dimitra Rontogianni, Stamatios Theocharis

**Affiliations:** 1 First Department of Pathology, National and Kapodistrian University of Athens, 11527 Athens, Greece; georgia.levidou@klinikum-nuernberg.de (G.L.); kpalamaris@yahoo.gr (K.P.); alexander.sykaras@gmail.com (A.G.S.); george-andr@hotmail.com (G.A.); cmasaout@med.uoa.gr (C.M.); theoirene@hotmail.com (I.T.); pkorkol@med.uoa.gr (P.K.); dgian@otenet.gr (D.R.); 2 Department of Pathology, Paracelsus Medical University, 90419 Nuremberg, Germany

**Keywords:** thymic epithelial neoplasms, thymomas, histones, CENP-A, HJURP, DAXX, histone modifications, histone variants

## Abstract

Background: Recent advances demonstrate the role of chromatin regulators, including histone variants and histone chaperones, in cancer initiation and progression. Methods: Histone H3K4me3, histone variant centromere protein (CENP-A) and histone chaperones Holliday junction recognition protein (HJURP) as well as DAXX expression were examined immunohistochemically in 95 thymic epithelial tumor (TET) specimens. Our results were compared with the expression profile of DAXX, HJURP and CENP-A in gene expression profiling interactive analysis (GEPIA2). Results: The lymphocyte-poor B3- and C-type TETs were more frequently DAXX negative (*p* = 0.043). B3 and C-Type TETs showed higher cytoplasmic and nuclear CENP-A (*p* = 0.007 and *p* = 0.002) and higher cytoplasmic HJURP H-score (*p* < 0.001). Higher nuclear CENP-A and cytoplasmic HJURP expression was associated with advanced Masaoka–Koga stage (*p* = 0.048 and *p* < 0.001). A positive correlation between HJURP and CENP-A was also observed. The presence of cytoplasmic CENP-A expression was correlated with a favorable overall survival (*p* = 0.03). CENP-A overexpression in survival analysis of TCGA TETs showed similar results. H3K4me3 expression was not associated with any clinicopathological parameters. Conclusions: Our results suggest a significant interaction between CENP-A and HJURP in TETs. Moreover, we confirmed the presence of a cytoplasmic CENP-A immunolocalization, suggesting also a possible favorable prognostic value of this specific immunostaining pattern.

## 1. Introduction

Over the past decades, epigenetic alterations have been established for their fundamental role in cancer initiation, progression, and metastasis [1]. In this context, accumulating evidence suggests that the structural adaptation of chromosomal regions to altered activity states and the misregulation of chromatin regulators, including histone variants, histone chaperones, modified histones, histone-modifying enzymes and effector proteins, as well as chromatin remodelers, can participate in tumorigenesis, tumor progression and metastasis [2,3]

Chromatin is organized in arrays of nucleosomes in which the core histones, H2A, H2B, H3 and H4, form an octameric core around which DNA is wrapped. The globular regions of the histones form the core of the nucleosome, while the N-terminal tails protrude from the nucleosomes and are enriched with a variety of posttranslational modifications. Histones are subject to a vast array of posttranslational modifications, including acetylation, methylation, phosphorylation, ubiquitylation, ADP-ribosylation, glycosylation and sumoylation [4]. Transcriptionally active and silent chromatin is characterized by distinct posttranslational modifications on the histones. Histone acetylation is primarily associated with gene activation, whereas methylation, depending on its position and state, can either be associated with repression or activation [5]. Active genes typically carry high levels of trimethylation of H3 lysine 4 (H3K4me3), trimethylation of H3 lysine 79 (H3K20me3), ubiquitylation of H2B on lysine 120 (H2Bk120ub1), and trimethylation of H3 lysine 36 (H3K36me3) [6]. On the other hand, marks associated with repressed genes include the trimethylation of H3 lysine 27 (H3K27me3), ubiquitylation of H2A on lysine 119 (H2AK129ub1), and trimethylation of H3 lysine 9 (H3K9me3) [6]. The chromatin-modifying enzymes that catalyze these marks, commonly called writers or erasers, can be recruited to target sites by sequence-specific DNA-binding transcription factors that regulate the transcriptional states of genes.

In addition to the major histone types, many histone variants have been found, originally characterized based on migration properties using Triton acid urea gel electrophoresis [7]. These histone variants either have a great similarity to the major histone types or present with an extremely divergent structure [8]. For H3 histones, several distinctive histone variants have been identified, the most characteristic of those being the replicative variant H3.1/2, the replacement variant H3.3 and the most divergent H3 variant CenH3 (centromeric histone 3), also called CENP-A (histone H3-like centromeric protein A) [9]. The latter is specifically associated with centromeric regions and shows a peak of expression during the G2/M phase of the cell cycle and the new CENP-A is incorporated during the late mitosis early G1 phase [10,11]. A particular group of histone chaperones, which act as dedicated architects, can selectively regulate the histone variant availability and placement across time and space in the nucleus. In this context, the histone chaperone Holliday junction recognition protein (HJURP) has been reported to be responsible for depositing CENP-A at the centromere in the late mitosis-early G1 phase [11,12,13], whereas DAXX/ATRX complex seems to contribute to the H3.3 presence at peri-centromeric regions and telomeres [14]. Moreover, a recent study showed that the experimental overexpression of CENP-A promotes ectopic deposition in the chromosome arms by the histone chaperone DAXX, instead of HJURP [14]. At these ectopic loci, CENP-A forms heterotypic nucleosomes, containing one each of the histone H3 variants, CENP-A and histone H3.3, within a single nucleosome [15].

Thymic epithelial tumors (TETs), namely thymomas and thymic carcinomas, comprise a heterogeneous group of neoplasms, not only in histological terms, but also in terms of biological behavior, which can range from an indolent to highly aggressive course [16]. TETs are categorized according to the WHO classification into types A, AB, B1-3 and C, based on the epithelial to lymphocyte ratio, the morphology of epithelial cells, and the similarity to normal thymus tissue [17,18]. The pathogenesis of most thymomas and thymic carcinomas remains unknown, largely precluding any targeted interventions [17]. According to the multiomics-based The Cancer Genome Atlas (TCGA) project, TETs exhibit an extreme paucity of targetable mutations [17]. On the other hand, there has recently been a great interest in the possible usage of immune checkpoint inhibitors (ICIs) targeting the programmed cell death protein-1 or programmed death-ligand 1 (PD-L1) as a therapeutical option for TETs, since they typically exhibit a PDL-1high phenotype [17]. At the same time, the research of epigenetic alterations in TETs has attracted the interest of medical oncologists and pathologists aiming to search for possible epigenetic biomarkers and to explore possible novel treatment options for these tumors [19]. In this context, numerous studies have reported the significant role of on non-coding RNA clusters and altered gene methylation in the tumorigenesis of TETs, which seem to be the most common epigenetic alterations in these tumors [19].

The expression of histone modifications, histone variants as well as histone chaperones in TETs and their potential role in tumorigenesis, tumor progression or patients’ prognosis, to our knowledge, has not been described. In view of the above considerations, the present study aims to evaluate the immunohistochemical expression of H3K4me3, which preferentially identifies active gene promoters, being one of the most investigated activation marks with a pivotal role in carcinogenesis, as well as histone variant CENP-A and histone chaperones DAXX and HJURP in TETs in association with clinicopathological parameters and patients’ survival.

## 2. Results

### 2.1. DAXX Expression and Associations with Clinicopathological Features

**Figure 1:**
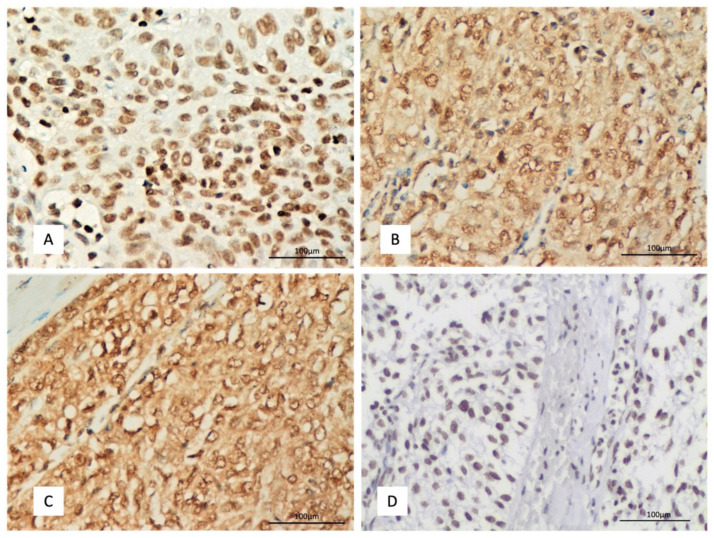
Immunohistochemical expression of (**A**) DAXX in a A type thymoma (**B**) CENP-A in a B3 type thymoma (**C**) HJURP in a B3 type thymoma and (**D**) H3K4me3 in a B3 type thymoma (×400).

COSMIC database and cBioPortal analysis do not reveal any DAXX mutation or genetic alteration in TETs TCGA cohort. DAXX expression was nuclear in both epithelial and lymphoid cells (Figure 1A). A positive immunoreactivity in epithelial cells was observed in 93.6% of the examined cases, with a median H-score of 90 and a range 0–300 (Table 1). Most these positive cases displayed a mild to moderate staining intensity (87%). Lymphocytic expression was moderate to strong in 86% of the cases. 

The lymphocyte-poor TETs, namely B3 and C, were more frequently negative for DAXX when compared to the rest TETs (Fischer exact test, *p* = 0.04, Figure S1). There were not any other differences between the other subtypes of TETs. 

DAXX expression either in the epithelial or in the lymphocytic component was not correlated with Masaoka–Koga stage, the presence of positive margins, with survival rates or other clinicopathological parameters (*p* > 0.10).

**Figure 2:**
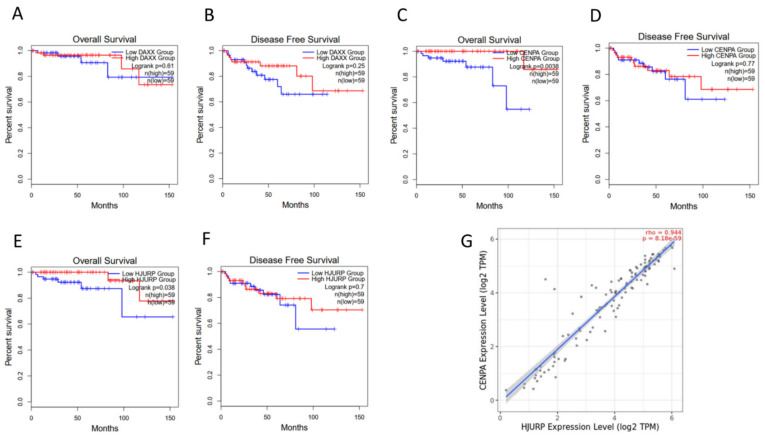
(**A**–**F**) TCGA TETs cohort survival analysis for DAXX expression (**A**,**B**), CENP-A (**C**,**D**) and HJURP (**E**,**F**). Survival analysis was performed with GEPIA2, using median as a cutoff. (**G**) TiMER2.0 analysis of TCGA TETs expression profile revealing a strong correlation between the expression of CENP-A and HJURP.

Survival analysis of the TCGA TETs cohort using GEPIA tool showed no association of DAXX expression with patients’ OS and DFS (Figure 2A,B).

### 2.2. CENP-A Expression and Associations with Clinicopathological Features

No mutations or copy number alterations of CENP-A were identified in the TETs TCGA cohort by using cBioPortal and COSMIC database. CENP-A expression was both cytoplasmic and nuclear in epithelial cells, 90% of the cases showing cytoplasmic and 39.8% of the cases nuclear CENP-A immunopositivity (Table 2, Figure 1B). None of the cases without cytoplasmic CENP-A immunoreactivity displayed a nuclear positivity, but there was a positive correlation between nuclear and cytoplasmic immunoreactivity (Spearman’s correlation coefficient, R = 0.37 *p* = 0.0002). CENP-A expression in lymphocytes was nuclear and was mainly mild to moderate in 85.5% of the cases.

**Figure 3:**
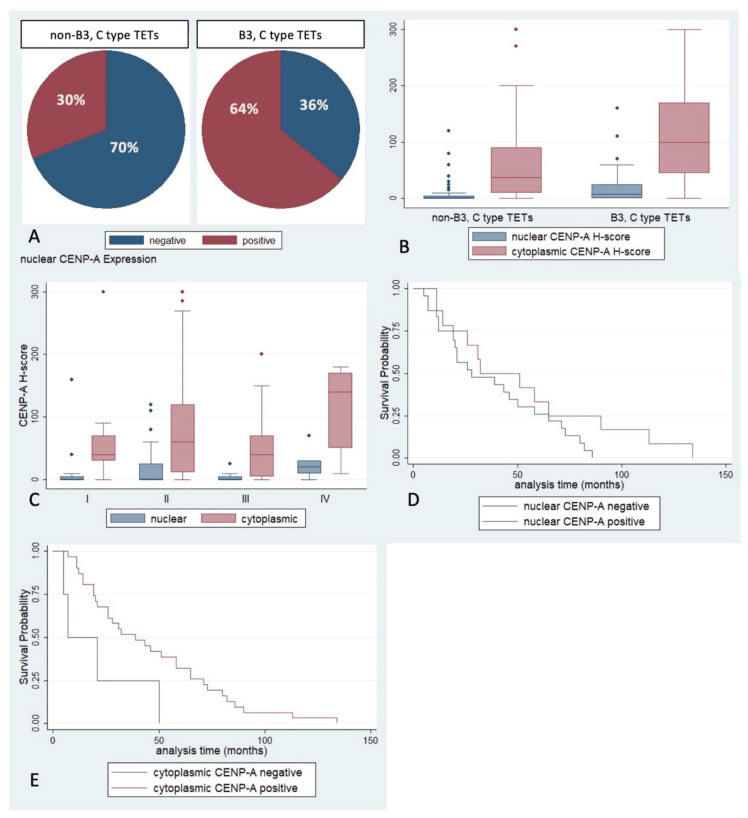
(**A**) Positivity rate of nuclear CENP-A in epithelial cells according to the WHO histological subtype (Fischer’s exact test, *p* = 0.01). (**B**) Schematic representation of the associations between CENP-A H-score and WHO subtype (Mann–Whitney U test, *p* = 0.01 for cytoplasmic and *p* = 0.02, for nuclear). (**C**) CENP-A H-score expression according to Masaoka–Koga stage (Mann–Whitney U test, IV vs. I/II/III, *p* = 0.04 for nuclear, *p* > 0.10 for cytoplasmic). (**D**,**E**) Kaplan–Meier survival rates according to nuclear (**D**) and cytoplasmic (**E**) CENP-A positivity (log-rank test, *p* > 0.10 for nuclear and *p* = 0.03 for cytoplasmic). Small diamonds in (**B**,**C**) correspond to outliers.

The lymphocyte-poor TETs, namely B3 and C, were more frequently positive for nuclear CENP-A (Fischer’s exact test, *p* = 0.01, Figure 3A) and showed higher cytoplasmic and nuclear CENP-A H-score (Mann–Whitney U test, *p* = 0.01 and *p* = 0.02 respectively, Figure 3B). There were no other differences between the other subtypes of TETs.

Moreover, higher nuclear CENP-A H-score was associated with advanced Masaoka–Koga stage (Mann-–Whitney U test, *p* = 0.04, Figure 3C), the advanced stage IV TETs showing a higher H-score when compared to the other categories. There was no significant association with the presence of relapse or the remaining clinicopathological parameters.

The presence of cytoplasmic CENP-A immunopositivity was correlated with a favorable overall survival (OS) in non-type C TETs (log-rank test, *p* = 0.03, Figure 3E). The median survival time for cases with cytoplasmic CENP-A expression was 39 months whereas the respective value for the cases which did not express CENP-A was 7 months. 

The expression of CENP-A in the lymphocytic component was not correlated with any of the clinicopathological parameters. 

TCGA TETs cohort survival analysis revealed that high expression of CENP-A is associated with better OS but not DFS (Figure 2C,D).

### 2.3. HJURP Expression and Associations with Clinicopathological Features

COSMIC database did not include any mutations of HJURP in TETs patients. On the other hand, cBioPortal identified the presence of HJURP alterations in 3/124 patients (2 type AB and 1 type A thymoma patients) from the TCGA TETs cohort. Specifically, these three patients had putative copy number alterations (deep deletions, homodeleted HJURP in all cases) whereas one of them with a type AB thymoma had five additional missense mutations of unknown significance. HJURP expression was mainly nuclear in epithelial cells (99% of the cases) with 69% of the cases showing also a cytoplasmic immunopositivity (Table 2, Figure 1C). The HJURP expression in lymphocytes was nuclear. Most of the positive cases displayed a mild to moderate nuclear and cytoplasmic immunoreactivity (84.7% and 95.3% respectively). Lymphocytic expression was, on the other hand, moderate to strong in 88% of the cases.

Cytoplasmic HJURP H-score was positively associated with nuclear and cytoplasmic CENP-A H-score (Spearman correlation coefficient, R = 0.34 *p* = 0.001 and R = 0.25 *p* = 0.016) and negatively with DAXX H-score (Spearman correlation coefficient, R = 0.21 *p* = 0.04).

**Figure 4:**
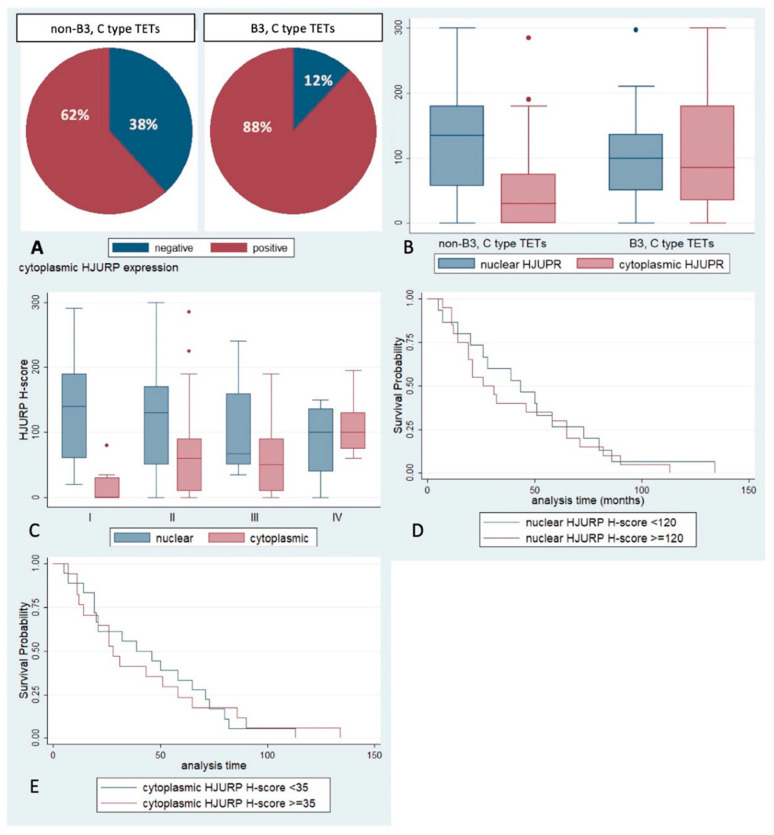
(**A**) Positivity rate of cytoplasmic HJURP in epithelial cells according to the WHO histological subtype (Fischer’s exact test, *p* = 0.02). (**B**) Schematic representation of the associations between HJURP H-score and WHO subtype (Mann–Whitney U test, *p* < 0.001 for cytoplasmic and *p* > 0.10 for nuclear). (**C**) HJURP nuclear and cytoplasmic H-score according to Masaoka–Koga stage (Mann–Whitney U test, *p* < 0.01 for cytoplasmic, *p* > 0.10 for nuclear). (**D**,**E**) Kaplan–Meier survival rates according to nuclear (**D**) and cytoplasmic (**E**) HJURP H-score (log-rank test, *p* > 0.10 for both associations). Small diamonds in (**B**,**C**) correspond to outliers.

The lymphocyte-poor TETs, namely B3 and C, were more frequently positive for cytoplasmic HJURP (Fischer’s exact test, *p* = 0.02, Figure 4A) and showed a higher cytoplasmic HJURP H-score (Mann–Whitney U test, *p* < 0.001, Figure 4B). There were not any other differences between the other subtypes of TETs.

Moreover, the presence of cytoplasmic HJURP expression was correlated with the Masaoka–Koga stage (Mann–Whitney U test, *p* < 0.01, Figure 4C), the majority of stage I tumors (64.7%) not displaying any cytoplasmic immunoreactivity for HJURP (Fischer’s exact test, *p* < 0.01). 

Nuclear HJURP expression in the epithelial and in the lymphoid cells did not show any correlation with histological subtype according to the WHO classification or with the Masaoka–Koga stage (*p* > 0.10). 

There was not any correlation of HJURP expression in the lymphoid or epithelial cells with survival rates (*p* > 0.10, Figure 4D,E).

TCGA TETs cohort survival analysis indicated that there is a correlation between overexpression of HJURP and a more favorable OS but not DFS (Figure 2E,F). Moreover, there is a strong correlation (Spearman correlation coefficient, R = 0.944 *p* < 0.0001) between CENP-A and HJURP expression in TCGA TETs as indicated by TIMER2.0 software analysis (Figure 2G).

### 2.4. H3K4me3 Expression and Associations with Clinicopathological Features

H3K4me3 expression was nuclear in both epithelial and lymphoid cells (Figure 1D). A positive immunoreactivity was observed in the epithelial cells in all the examined cases, showing a median H-score of 100 (Table 2, Figure 1D). The vast majority of the cases showed either a mild (44%) or a moderate (51%) staining intensity. The expression in the lymphoid cells was more often strong (69% of the cases). 

H3K4me3 expression was positively correlated with nuclear and cytoplasmic CENP-A H-score (Spearman’s correlation coefficient, R = 0.33, *p* = 0.001 for nuclear and R = 0.24, *p* = 0.02 for cytoplasmic CENP-A). There was no significant association between H3K4me3 H-score and HJURP or DAXX H-score (*p* > 0.10).

**Figure 5:**
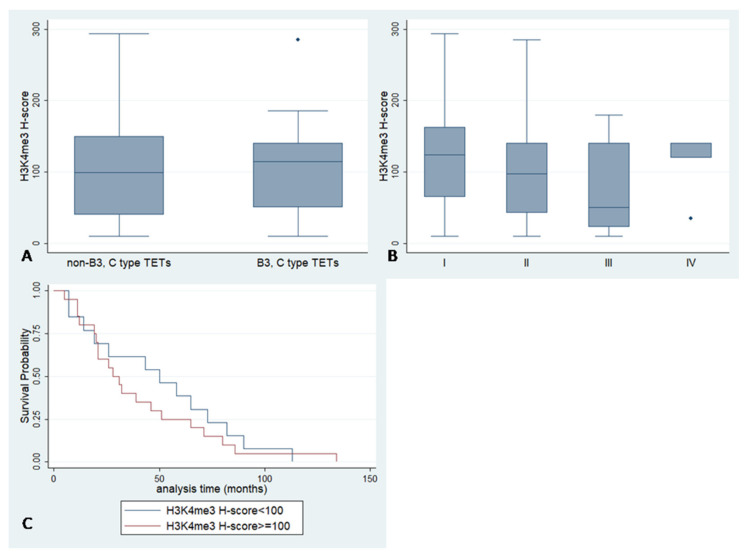
(**A**,**B**) H3K4me3 Histone H-score according to WHO subtype (Mann–Whitney U test, *p* > 0.10) and Masaoka–Koga stage (Kwallis ANOVA, *p* > 0.10) (**C**) Kaplan–Meier survival curves according to H3K4me3 H-score (log-rank test, *p* > 0.10). Small diamonds in (**A**,**B**) correspond to outliers.

H3K4me3 expression either in the epithelial or in the lymphocytic component was not correlated with subtype according to the WHO classification, Masaoka–Koga stage, the presence of positive margins, with survival rates or other clinicopathological parameters (Figure 5, *p* > 0.10).

## 3. Discussion

TETs represent the most common tumors of the thymus gland. Currently, the standard first line therapy for early-stage detectable TET is thymectomy. More advanced stage tumors are treated with chemotherapy after surgery, especially in the case of incomplete tumor resection [19]. Several studies have tried to evaluate the role of potential biomarkers in TETs, aiming also to identify potential therapeutic targets in these tumors [19]. Aberrant epigenetic alterations seem to be involved in the pathogenesis of thymomas and thymic carcinomas [19]; however, the possible role epigenetic regulators, such as histone variants, histone modifications and their related chromatin remodelers remains largely unexplored. In this aspect, the present investigation studies for the first time the immunohistochemical expression of histone modification H3K4me3, histone variant CENP-A, as well as chaperones HJURP and DAXX in TETs.

In this study, the immunohistochemical expression of CENP-A, HJURP and DAXX was observed in the epithelial cells in most of the investigated cases. In this context, the positivity rate for CENP-A was 90%, for HJURP 99% and for DAXX 93.6%. The overexpression of CENP-A is one of many factors implicated in promoting chromosomal instability and has been reported in several human malignancies, including hepatocellular [20], colorectal [21], breast carcinoma [22] as well as lung [23] and ovarian adenocarcinoma [24]. Similarly, the upregulation and overexpression of HJURP has been observed in lung [25], ovarian and prostatic adenocarcinoma [26], and in breast carcinoma [3], as well as in gliomas [27]. The same applies to DAXX which has been reported to be overexpressed in a great variety of human malignancies (reviewed in [28]). 

Interestingly, we observed a predominant cytoplasmic immunoreactivity for CENP-A and a nuclear and cytoplasmic immunoexpression for HJURP. The subcellular localization of these proteins was initially reported to be the nucleus, in keeping with their function in the regulation of chromatin. In line with our findings, HJURP expression has been reported in prostate cancer to be predominantly cytoplasmic [26,29], the hepatocellular carcinomas also display a cytoplasmic expression [30,31], whereas in gliomas, HJURP immunoreactivity is observed both in the nucleus and in the cytoplasm [32]. On the other hand, the expression in other types of carcinomas, such as pancreatic, colorectal, lung and ovarian, was nuclear [23,24,33,34]. These findings suggest that the subcellular localization of HJURP varies in different types of human malignancies. Moreover, a recent study reported different patterns of subnuclear CENP-A localization among cases with head and neck carcinomas, a specific CENP-A immunoreactivity at the nuclear periphery being able to predict locoregional disease control after chemoradiation [35]. The predominantly cytoplasmic immunoexpression of CENP-A has not been previously reported. Recent studies have reported the presence of two motifs in the amino-terminus of CENP-A, namely R42R43R44 and K49R52K53K56, which also seem to be required for DNA contact in the centromere nucleosome and are critical for CENP-A accumulation in the nucleus [36]. According to these observations, these motifs seem to be required but are not sufficient for the nuclear import of CENP-A, whereas other elements may participate in this purpose [36]. The different subcellular localization of these molecules in cancer cells may lead to different functions, but the specific mechanisms require further investigation.

In our study, the immunohistochemical expression of H3K4me3 was nuclear and was observed in all the examined cases (100%). A similar nuclear pattern of expression has been reported in hepatocellular [37] and breast carcinoma [38], whereas cervical carcinoma shows both a cytoplasmic and a nuclear pattern of immunoreactivity [38]. High levels of H3K4me3 have been reported in several type of neoplasms, such as in human lung carcinoma cells as wells as in cervical, breast and hepatocellular carcinoma [37,38,39,40]. On the other hand, leukemia cells and bladder cancer cells have been reported to express low levels of H3K4me3 [41]. Moreover, H3K4me3 immunohistochemical expression has been correlated with a poor prognosis in hepatocellular, cervical and breast carcinoma [37,38,40], whereas it did not convey any prognostic significance in renal cell carcinoma [42]. Although in our study, H3K4me3 expression was observed in all cases did not find any association with the clinicopathological parameters or patient’s overall survival. It is already known the trimethyl form, H3K4me3, preferentially identifies the gene promoters that are active, promoting a decondensed (“open”) configuration of chromatin which allows transcription factors access to binding sites [38]. However, it seems that each cancer type has a different expression pattern of H3K4me3, which could account for a different role in the course of each tumor type.

An interesting finding of the present investigation is that there was a different pattern of CENP-A and HJURP expression in lymphocyte rich types of TETs when compared to the lymphocyte poor (namely B3 and C type) TETs. Interestingly, both nuclear and cytoplasmic CENP-A, as well as cytoplasmic HJURP, were higher in B3 thymomas and thymic carcinomas. Moreover, both CENP-A and HUJRP were associated with Masaoka-Koga stage, which remains an established prognostic parameter for thymic neoplasms [17]. Nuclear CENP-A expression was in our study higher in stage IV TETs, whereas the vast majority of stage I TETs did not display any expression of HJURP. Recent studies have documented that CENP-A overexpression promotes aneuploidy with karyotypic heterogeneity contributing to an aggressive phenotype in CENPA overexpressing cancers [43]. Since similar associations of CENP-A and HJURP with tumor stage have been previously reported in a variety of carcinomas. Increased HJURP expression has been correlated advanced with tumor stage in prostate cancer and ovarian adenocarcinoma [20,26], whilst increased CENP-A expression has been associated with advanced tumor stage in lung and ovarian adenocarcinomas [23,24]. These findings provide evidence that both CENP-A and HUJRP could be implicated in the pathogenetic mechanisms underlying the neoplastic evolution in the thymus gland. 

Furthermore, we found a significant positive correlation between nuclear or cytoplasmic expression of CENP-A and cytoplasmic HJURP. Gene expression analysis of the TCGA TETs also revealed a strong correlation between CENP-A and HJURP expression, which further implicates the possible interaction between these molecules during oncogenesis in thymus gland. The role of the histone chaperone HJURP in the deposition of CENP-A at the centromere is well known, and previous studies have demonstrated that knockdown of HJURP in cancer cells strongly affects not only CENP-A deposition but also centrosomes and chromosome stability [26]. A complex of CENP-A/H4 heterodimers in association with HJURP is targeted to centromeres in telophase/early G1 to allow deposition of CENP-A/H4 during the G1 phase [44]. CENP-A is also deposited at sites of high histone turnover outside of the centromere DNA. This ectopic deposition is increased when CENP-A is overexpressed [43]. However, the exact mechanisms by which the interaction of CENP-A with HJURP can potentially promote oncogenesis and disease progression remain to be fully elucidated.

Although nuclear CENP-A immunoexpression was correlated with advanced tumor stage, we also found that cytoplasmic CENP-A expression was a significant marker of favorable prognosis in our cohort. This is the first study to correlate an overexpression pattern of CENP-A with better patients’ OS. Similar to our study, the survival analysis of TCGA TETs reveals that the overexpression of CENP-A is associated with better prognosis. A limitation of our study in this context is that survival data were available in a small subgroup of our cohort, in which we were able to perform survival analysis. However, univariate survival analysis in this subgroup was able to recapitulate a significant prognosticator of TETs, namely the Masaoka–Koga stage, suggesting that it is representative. Several studies have previously investigated the potential prognostic role of CENP-A expression in other tumor types, such as lung and ovarian adenocarcinomas, and presented nuclear CENP-A expression as a marker of worse prognosis [23,24]. The cytoplasmic localization of CENP-A can been explained in the absence of the specific motifs, its amino-terminus or other necessary molecules, which are necessary for its nuclear transition [36]. It could be hypothesized that different subcellular localization of CENP-A may lead to different functions, but the specific mechanisms, however, explaining the possible role of this cytoplasmic localization CENP-A require further investigation. 

In our study, DAXX expression showed an opposite pattern of expression compared to CENP-A and HJURP in different histological types of TETs. In this context, the lymphocyte poor TETs, namely thymomas B3 and thymic carcinomas, showed a decreased expression of DAXX, which was predominant in the rest type of TETs, possibly having a role in the evolution of these tumors. Moreover, we found a negative correlation between the cytoplasmic HJURP expression and DAXX expression. Histone chaperone DAXX was initially supposed to be a dedicated architect of histone variant H3.3, being responsible for its localization in peri-centromeric regions and telomeres [14]. Some studies also implicated a potential role of DAXX in the ectopic deposition of CENP-A in the chromosome arms, where it forms heterotypic nucleosomes [15]. In our study, however, we did not find any significant association between CENP-A and DAXX expression, and the DAXX H-score was not correlated with any of the remaining clinicopathological parameters. Additional data from the TCGA also indicate that DAXX expression is not associated with TET patients’ survival.

In our study, we also found an overexpression of all the investigated molecules in the lymphocytic component of the tumors, with a positivity rate being in all cases higher than 95%. However, there was not any correlation of the expression of CENP-A, HJURP, DAXX or H3K4me3 in the lymphocytic component of TETs with any of the investigated clinicopathological parameters, such as histological subtype, Masaoka–Koga stage or with patient’s OS. The lymphocytic component of TETs belongs to the tumor microenvironment and has received great research attention in the last years, with studies investigating either its role in the tumorigenesis or its potential therapeutic targeting [45]. 

## 4. Materials and Methods

### 4.1. Patients

This is a study of archival formalin-fixed paraffin-embedded (FFPE) tissue from 95 patients with TETs resected between 2009 and 2019, retrieved from the pathology laboratory archives of two major hospitals in Athens, Greece (Evangelismos General Hospital and Laikon General Hospital), for whom medical records were available. Patient characteristics are shown in Table 2. Forty-two of the patients were men (44.2%) and 53 women (55.8%), with a median age at diagnosis 63 years (range 27–88 years). The frequency of WHO subtypes was as follows: type A 11.6%; type AB 23.2%; type B1 17.9%; type B2 19%; type B3 13.7%; micronodular thymoma with lymphoid stroma (MNT) 2%; type C 12.6%. Masaoka–Koga stage was I in 20%; IIa in 40%; IIb in 16.5%; III in 16.5%; IVa in 3.5%; IVb in 3.5% of patients. Surgical margins were positive in 28.3% of cases. Co-existing myasthenia gravis was diagnosed in 58.9% of patients, 2 of whom also suffered from pemphigus vulgaris and autoimmune thyroidopathy; among the patients without myasthenia gravis, 1 had pure red cell aplasia, and 1 had hypothyroidism. Chemotherapy was given to 27% and radiotherapy to 50% of patients for whom respective information was available; 5 of these patients received both chemo- and radiotherapy. Follow-up information was available for 38 patients, ranging from 5 to 134 months (median: 32 months). 

### 4.2. TMA Construction

One representative FFPE tissue block from each tumor was selected after all hematoxylin–eosin (H&E)-stained slides were reviewed. TMAs were constructed using a manual tissue using a manual tissue arrayer (TMA Model I, Beecher Instruments, Sun Prairie, WI, USA). More specifically, three to five 1.5 mm cores were transferred from each representative block into positionally encoded arrays in eight recipient paraffin blocks. Multiple cores from each single case were included to capture histological tumor heterogeneity. Tonsils, placenta, and normal kidney tissue were used as controls during the construction of the TMAs. 

### 4.3. Immunohistochemistry

Immunohistochemistry was carried out using standard procedures in the eight TMAs. The sections were stained with antibodies against DAXX (clone ab239806, AbCam, Cambridge, UK, at dilution 1:50), HJURP (clone ab100800, AbCam, at dilution 1:5000), CENP-A (clone Ab217622, AbCam, at dilution 1:200) and H3K4me3 (clone C4C8, Cell Signaling, at dilution 1:1500). Antigen retrieval was performed at pH 6. The Envision (Dako, Agilent, Santa Clara, CA, USA) visualization system was used. DAB (3,3-diaminobenzidine) was used as a chromogen, and hematoxylin as a counterstain. Appropriate positive controls according to the manufacturer were used. As a negative control, the omitted primary antibody and substitution with an irrelevant antiserum was used. 

For the purposes of the immunohistochemical evaluation, we calculated the H-score, which serves as a semiquantitative measure of the immunohistochemical protein expression levels. To calculate the H-score, the semiquantitative staining intensity score (score 1 to 3) is multiplied by the percentage of positive cells. Therefore, H-score values range between 0 and 300. The epithelial and the lymphocytic components, as well as the nuclear and cytoplasmic positivity, were separately evaluated.

### 4.4. Database and TCGA Analysis

#### 4.4.1. Gene Expression Profiling Interactive Analysis (GEPIA) 

The expression profile of DAXX, HJURP and CENP-A in TETs was analyzed with GEPIA2. GEPIA2 is a database that retrieves data from TCGA tumor samples and adjacent genotype-tissue expression (GTex) normal samples [46]. Moreover, the survival analysis module of the GEPIA2 database was used to analyze the association between the expression of DAXX, HJURP and CENP-A and patients’ overall and disease-free survival (OS and DFS). A median cutoff value (50%) was employed to generate Kaplan–Meier curves, and the log-rank *p*-value was calculated.

#### 4.4.2. Tumor Immune Estimation Resource (TIMER2.0) Analysis

We used the Gene Corr module of TIMER2.0 to investigate the correlation between HJURP and CENP-A expression in TETs. TIMER2.0 is a web resource for TCGA tumors gene expression analysis and immune infiltrate profiling [47].

#### 4.4.3. Genetic-Copy Number Alteration and Mutation Analysis

We assessed the Catalogue of Somatic Mutations in Cancer (COSMIC) database v96 (released 31 May 2022) [48] and the cBioPortal for Cancer Genomics [49] to check for mutations or genetic-copy number alterations of DAXX, CENP-A and HJURP in TETs TCGA patients.

### 4.5. Statistical Analysis

Statistical analysis was performed by a MSc biostatistician (GL). The association between the IHC expression of DAXX, CENP-A, HJURP and H3K4me3 with clinicopathological characteristics was examined using non-parametric tests with correction for multiple comparisons, as appropriate. Survival analysis was performed using Kaplan–Meier survival curves, and the differences between the curves were compared with log-rank test. A *p*-value of <0.05 was considered statistically significant. The analysis was performed with the statistical package STATA 11.0/SE (College Station, TX, USA) for Windows.

## 5. Conclusions

In conclusion, our study suggests for the first time a possible association between the overexpression of histone variant CENP-A and its dedicated chaperone HJURP in the evolution and progression of TETs, being associated with lymphocyte-rich tumors and advanced tumor stage. On the other hand, chromatin remodeler DAXX was preferably expressed in non-B3 and C TETs. H3K4me3, though constitutively present in TETs, seems to play a less informative role in this regard. Further studies involving also other chromatin regulators are necessary in order to validate and expand our results. Understanding the complexity of the participation of several chromatin epigenetic alterations in these tumors not only can elucidate their implication in oncogenesis, but also can lay the foundation for the development and application of novel therapeutic agents for this rare type of malignancy.

## Figures and Tables

**Table 1:** Expression of DAXX, CENP-A and HJURP in TETs.

	Positivity Rate	H-Score, Median	H-Score, Range
**Epithelial cells**			
DAXX nuclear expression	93.6%	90	0–300
CENP-A cytoplasmic expression	90%	50	0–300
CENP-A nuclear expression	39.8%	0	0–160
HJURP cytoplasmic expression	69%	35	0–300
HJURP nuclear expression	99%	120	0–300
H3K4me3 nuclear expression	100%	100	10–294
**Lymphoid cells**			
DAXX	95.7%	200	0–300
CENP-A cytoplasmic expression	98.6%	140	0–300
HJURP nuclear expression	98.3%	235	0–300
H3K4me3 nuclear expression	100%	270	0–300

**Table 2:** Clinicopathological characteristics of 95 patients with TETs.

Parameter	Median	Range
**Age**	63	27–88 years
	**Number**	**%**
**Gender**		
Male	42/95	44.2%
Female	53/95	55.8%
**WHO subtypes**		
Type A	11/95	11.6%
Type AB	22/95	23.2%
Type B1	17/95	17.9%
Type B2	18/95	19.0%
Type B3	13/95	13.7%
Micronodular with lymphoid stroma	2/95	2%
Type C	12/95	12.6%
**Masaoka–Koga stage**		
I	17/85	20%
IIa	34/85	40%
IIb	14/85	16.5%
III	14/85	16.5%
IVa	3/85	3.5%
IVb	3/85	3.5%
**Positive surgical margins**	13/46	28.3%
**Presence of myasthenia Gravis**	33/56	58.9%
**Presence of chemotherapy**	10/37	27%
**Presence of radiotherapy**	18/36	50%
**Event**		
Alive disease free	25/38, follow-up 5–134 months	71.4%
Alive with disease	3/38, follow-up 28–65 months	8.6%
Dead of disease	7/38, within 11–65 months	20%
**Presence of relapse**	3/33, within 58–65 months	9.1%

## Data Availability

The data presented in this study are available on request from the corresponding author.

## Supplementary Materials

The supporting information can be downloaded at: https://www.mdpi.com/article/10.3390/ijms23158339/s1.
