# Supplementary material for: Unraveling the Role of Histone Variant CENP-A and Chaperone HJURP Expression in Thymic Epithelial Neoplasms
Source: Int J Mol Sci. 2022 Jul 28;23(15):8339. doi: 10.3390/ijms23158339 (PMC9368969; doi:10.3390/ijms23158339)

Figure S1. (**A**) Positivity rate of DAXX in epithelial cells according to the WHO histological subtype (Fischer exact test, $p = 0.04$). (**B**) DAXX H-score according to Masaoka-Koga Stage (Kwallis ANOVA, $p > 0.10$). (**C**) Kaplan-Meier survival curves according to DAXX H-score (log-rank test, $p > 0.10$).

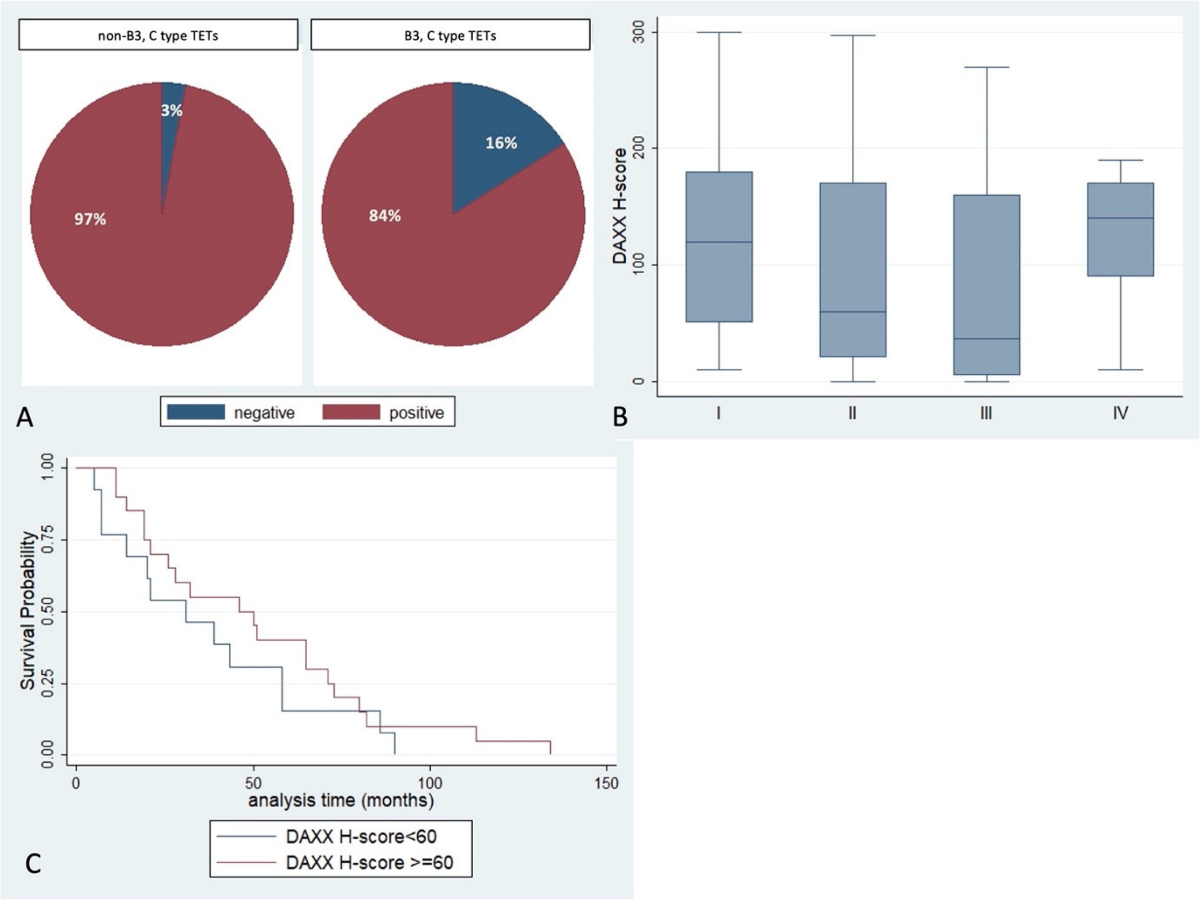

Supplement: Supplementary file 1 [file ijms-23-08339-s001.zip › ijms-1816122-supplementary.pdf]
